# Mercury

**Published:** 1890-06

**Authors:** Adolph Lippe


MERCURY causes, among the workers in it, an immunity from syphilis. It also causes carious teeth, salivation, spongy gums. It cures both homœopathically. It causes tremor of the hands among these workers like the tremor we find in drunkards; it also cures it.—*Extract from a letter of the late Dr. Adolph Lippe.*

